# Examining Factors Influencing Cognitive Load of Computer Programmers

**DOI:** 10.3390/brainsci13081132

**Published:** 2023-07-28

**Authors:** Didem Issever, Mehmet Cem Catalbas, Fecir Duran

**Affiliations:** 1 Department of Computer Engineering, Faculty of Technology, Gazi University, 06560 Ankara, Turkey; fduran@gazi.edu.tr; 2 Department of Electronics and Automation, 1st Organized Industrial Zone Vocational School, Ankara University, 06935 Ankara, Turkey; mccatalbas@ankara.edu.tr

**Keywords:** saccadic eye movement, cognitive load, eye tracking, computer programming, canonical correlation, linguistic distance

## Abstract

In this study, the factors influencing the cognitive load of computer programmers during the perception of different code tasks were investigated. The eye movement features of computer programmers were used to provide a significant relationship between the perceptual processes of the sample codes and cognitive load. Thanks to the relationship, the influence of various personal characteristics of programmers on cognitive load was examined. Various personal parameters such as programming experience, age, native language, and programming frequency were used in the study. The study was performed on the Eye Movements in Programming (EMIP) dataset containing 216 programmers with different characteristics. Eye movement information recorded during two different code comprehension tasks was decomposed into sub-information, such as pupil movement speed and diameter change. Rapid changes in eye movement signals were adaptively detected using the *z*-score peak detection algorithm. Regarding the cognitive load calculations, canonical correlation analysis was used to build a statistically significant and efficient mathematical model connecting the extracted eye movement features and the different parameters of the programmers, and the results were statistically significant. As a result of the analysis, the factors affecting the cognitive load of computer programmers for the related database were converted into percentages, and it was seen that linguistic distance is an essential factor in the cognitive load of programmers and the effect of gender on cognitive load is quite limited.

## 1. Introduction

Saccadic eye movements are quick movements that occur when visual focus on an object changes rapidly [1]. There are strong connections between saccadic eye movements and cognition levels. It has been found that it is meaningful to analyze not only saccadic eye movements but also pupillary parameters to determine cognitive states; with suitable combinations of these variables, the cognitive load levels of individuals for specific activities can be determined [2]. Therefore, many studies have analyzed the cognitive levels of individuals using eye movements for different tasks [3]. The measurement of rapid eye movements, such as saccadic eye movements, has improved with the performance of eye-tracking devices, and the success and number of studies in this field have increased significantly in recent years. In this article, some of these studies are discussed. Pfleging et al. conducted a fully controlled experiment with an intuitive classifier under different illumination conditions, during which a dataset and preliminary model were developed to estimate the mental load level based on eye movement [4]. In a driving simulator study, Kun et al. analyzed the pupil diameter measurements of a distant speaker and a driver preparing to speak and discovered that the pupil diameter of the driver was more significant than that of the remote speaker as the driver was preparing to speak. They also investigated the effects of different conversations on the pupil diameter. Consequently, they aimed to create a dialogue system capable of adapting to the driver’s behavior under a high cognitive load [5].

Kruger and colleagues used the percentage change in pupil diameters to examine the cognitive load that occurs when participants watch a video with and without subtitles. They discovered cognitive load decreased in courses with subtitles when watching videos [6]. Chen et al. suggested an intelligent cognitive load monitoring system based on the use of the eyes. According to the proposed method, the cognitive load was created using arithmetic exercises and controlled by the number of moves and digits. They used regression models to identify more than two cognitive load levels using different eye activity models. It was observed that the pupil diameter and number of blinks were found to increase more during complicated tasks [7]. Zagermann et al. proposed employing eye-tracking tools to study users’ cognitive load when interacting with a system. In this context, they provided a model that integrates human–computer interaction features into the link between eye-tracking data and cognitive load. They believe that this approach will help develop interfaces that require less cognitive capacity and suggest that eye tracking is a significant tool for examining cognitive processes in visual computation [8]. In another study, Cong et al. examined the relationship between cognitive load and eye movements. They investigated cognitive load in the multimedia presentation process. They developed a quantitative model to assess cognitive load in terms of knowledge comprehension and used pupil diameter variation for an assessment focused on the cognitive load theory [9]. 

The term cognition refers to the ability of the brain to collect, process, and convert information received from various sources such as sensory stimuli, perception, experience, and vision. Cognitive load theory aims to objectively analyzes and make sense of the human brain’s perception, understanding, and learning processes [10]. This exciting theory is used in many disciplines, especially education. Although it is believed that there are evolutionary similarities in information processing in individuals, it should be remembered that the ability to store information varies from person to person and is influenced by a variety of factors [11]. John Sweller developed cognitive load theory (CLT), and an article on the subject was published in the journal Cognitive Science in 1988 [12]. John Sweller revised this theory in an article published in 2019 owing to developments in the theory over time. CLT aims to aid scientists in developing unique instructional solutions compatible with the limitations of the human cognitive system. Working memory, or short-term memory, has a finite capacity and can only effectively process a limited amount of information instantly. This assumption regarding memory capacity for processing information is the basis of the cognitive load theory. Cognitive load theory aims to build meaningful learning design concepts that focus on the human cognitive system. According to cognitive load theory, human cognitive architecture assumes cognitive schemes that contain limited working memory and unlimited long-term memory; expertise is obtained only from knowledge stored in long-term memory as structures. Cognitive load is a multidimensional concept that affects a learner’s cognitive system while performing a task. The framework of Paas and van Merrinboer’s broad CLT model contains a causal dimension that reflects the interaction of task and learner characteristics and an evaluative dimension that reflects the concepts of measurable load, effort, and mental performance [13,14]. Every stimulus the individual is exposed to while in the learning position creates a load on the individual’s mind, and these stimuli may be in the environment in a desired or undesirable manner.

According to CLT, there are three types of cognitive load: intrinsic, extraneous, and germane [15]. Intrinsic load is a cognitive load produced by the complexity or difficulty of learning new knowledge. Researchers in this field have found that learning a structure with many interaction components is more complex than learning a system with fewer interaction elements and requires more cognitive capacity to process [16]. The learning technique cannot change the intrinsic load; it can only change with the degree of skill. Expert learners with extensive knowledge can quickly combine complex information items with pre-existing schemas and manipulate schema development as a working memory item. Therefore, individuals who are experts in their field have a low intrinsic load, despite solving complex problems. According to CLT, there are two types of loads apart from the intrinsic load [17]. Extraneous load refers to the mental resources allocated to objects that do not contribute to learning and schema creation. This part of cognitive load is about the presentation of information and the instructional format, which can increase the total cognitive load while having little effect on learning; it is the amount of memory consumed by all the hidden programs operating in the background in the system tray [18]. Germane load represents partitioned mental resources for creating and organizing long-term memory schemas, and it is similar to memory usage when loading a program on a computer. In this context, germane load defines the activity of long-term storage of information or schemas, and the peculiarity of this section significantly accelerates the learning process. Germane load enables the development of cognitive behavioral patterns necessary to make sense of the categories of information. As a result of the aggregation of these three loads, cognitive load emerges. This also means that the total cognitive load an individual experiences in working memory while performing a specific task is the sum of the three different load types.

**Figure 1:**
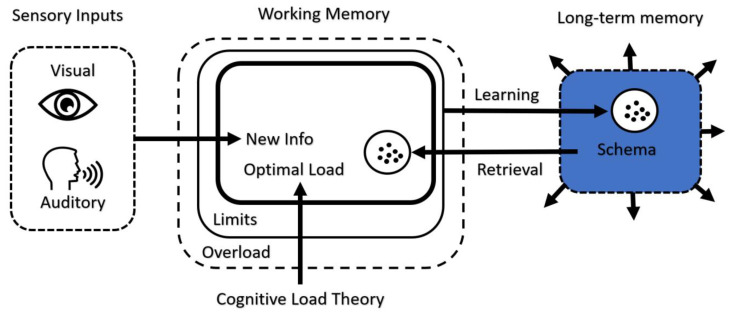
CLT and schema representation.

Learning is connected to increasing the processing capacity of the working memory. The learning process involves transferring information from working memory to long-term memory. As in Schema theory, this information transmission allows information to be structured as a schema in long-term memory. Creating a schema entails connecting disparate pieces of information to move from a lower to a higher level of complexity and keeping them together as a single meaningful whole chunk of information. Figure 1 illustrates a mental architecture model and the function of CLT in working memory and schema formation [18].

Measuring this load is as important as defining the concept of a cognitive load. Many methodologies and measurement tools can be used to determine the cognitive load. The following are some approaches for evaluating cognitive load frequently utilized in the literature.

- Emotional Scales: Participants are administered questionnaires assessing the difficulty and confusion level of cognitive tasks.
- Performance Measures: Performance measures such as the time to complete a task, level of accuracy, or number of errors can be indicators of cognitive load.
- Psychophysiological Measures: Physiological measurements such as electroencephalogram (EEG), electrocardiogram (ECG), eye movements, or skin conductance can be used to determine cognitive load.

Among these approaches, studies using physiological measurements have increased in popularity in recent years due to their avoidance of subjectivity and the development of sensor technologies. Eye-tracking systems stand out among various physiological parameters for estimating cognitive load. There are various reasons for this. The stimulus is usually presented to the user visually, with the result that there is a significant change in eye movements; there is no physical connection between eye tracking systems and the user; eye movements can be interpreted more easily than other physiological signal measurements; the cost of an eye-tracking system is as much as a comprehensive EEG device or magnetoencephalography (MEG) device; and its application does not require any pre-processing that requires expertise. There are many studies on the calculation of cognitive load from eye movements. In a study conducted in this context, pupil responses were used to establish a meaningful relationship between cognitive load and the difficulty level of a video game [19]. In another study that examined the behavior of map users in the process of performing specific tasks using the relationship between cognitive load and eye movements, significant differences were observed between the eye movements of experts and novice users. As a result of the study, novices were observed to have longer fixation durations and higher saccade velocity, which indicates a higher cognitive load for novices [20]. Another comprehensive study in this field also found a positive correlation between pupil dilation and cognitive load [21]. In another study, the effects of visual and auditory stimuli on cognitive load were analyzed using eye movements. In a related study, the impact of background music (BGM) on cognitive load in learning processes was analyzed through eye movements. It was found that listening to BGM imposed a higher cognitive load on post-lexical processes than on lexical processes [22]. Eye movement and pupillary response indicators of cognitive load, commonly used in studies, are listed below.

- Pupillary diameter mean
- Pupillary diameter deviation
- Saccadic eye movement
- Pupillary hippus

Saccadic eye movement is a type of rapid eye movement in which the eye shifts rapidly from one focal point to another. It is an essential component of visual perception and is controlled by a complex network of neuronal circuits in the brain [23]. During a saccade, the eyes move rapidly from one point to another, followed by a brief fixation that allows the brain to interpret visual information. Because saccadic eye movement is a form of eye movement at high speeds, observing with cameras with high sampling rates is more meaningful and efficient. Saccadic eye movements are essential in various tasks and have been extensively studied in cognitive psychology, neurology, and ophthalmology. 

The main objective of this research is to identify the variables that influence programmers’ cognitive load during computer programming activities. An extensive database collecting quantitative and categorical information on programmers’ spoken language, programming experience, and age was used. Through linguistic distance, categorical information in the database, such as native language, was transformed into quantitative variables, and the database of 216 participants was made appropriate for study through numerical values. As a result of the study, the factors influencing the cognitive load of programmers were determined and detailed in percentages.

## 2. Materials and Methods

A multivariate analysis method was required to develop the necessary mathematical model within this study’s scope. Multivariate analysis methods create a logical relationship between datasets that contain many observations or variables [24]. Creating a meaningful pattern among this large number of variables provides an efficient mathematical model for solving problems and understanding the main pattern of the dataset. Feature extraction, economics, medicine, and pharmacy sciences are a few application areas of multivariate analytic analysis techniques [25,26]. Because the dataset produced in this study contains numerous variables, an effective multivariate analysis method should be implemented. Therefore, the canonical correlation analysis approach was selected, which is frequently used in multivariate analysis methods and is helpful in understanding and evaluating their outputs.

### 2.1. Canonical Correlation Analysis

Canonical correlation analysis (CCA) is an effective multivariate analysis tool for determining and evaluating the relationship between two multivariate variable sets [27]. Additionally, it can be used as a dimension-reduction application, such as a principal component or linear discriminant analysis. The main aim of CCA is to discover canonical coefficients that maximize the linear relationships between two datasets [28]. H. Hotelling proposed and published CCA in the literature in 1936, and it has since been used in statistics, biometrics, meteorology, epidemiology, and many other disciplines [29]. CCA is often used in various sectors, including medicine, education, economics, and engineering, to explore and comprehend the relationships between observations. It will be utilized in this study to analyze the importance of the relationship between two different datasets as well as the ratio of the sub-components that influence this relationship. One of these datasets contains cognitive load factors, while the other includes programmers’ personal parameters. The basic form of multivariate regression analysis is performed using the coefficient values that provide the best linear relationship between the input parameters *x*_1_, *x*_2_, …, *x_i_* and the single variable *y*. The CCA approach examines this relationship over a large number of inputs (*x*_1_, *x*_2_, …, *x_i_*) and outputs (*y*_1_, *y*_2_, …, *y_j_*), which is performed with the help of parameters consisting of the most suitable coefficients that would maximize the correlation between these two multivariate datasets [30]. 

Canonical variables are parameters that optimize the correlation between these two datasets and define the best linear relationship according to each other. The coefficients that enable this linear and optimal relationship to be established according to the input data are defined as the canonical weights. The relationship between input datasets, canonical weights ($b_{i},\;a_{i}),$ and canonical variables (*V_i_, U_j_*) is given in Equations (1) and (2).

(1) $$V_{1}=b_{1}x_{1}+b_{2}x_{2}+b_{3}x_{3}+\cdots +b_{i}x_{i}$$

(2) $$U_{1}=a_{1}y_{1}+a_{2}y_{2}+a_{3}y_{3}+\cdots +a_{i}y_{i}$$

In the CCA approach, canonical variables are obtained depending on the size of the dataset, and the canonical correlation coefficient (*R*) values are obtained in relation to these variables. The eigenvalue problem in Equations (3) and (4) needs to be solved to obtain the canonical variables that are important for canonical correlation and to provide a linear relationship between the datasets. *X* and *Y* represent the datasets which are used in canonical correlation analysis, and $C_{xy}$ is the covariance between them.

(3) $$R=\frac{E\left[U_{x}V_{y}\right]}{E\left[{U_{x}}^{2}\right]E\left[{V_{y}}^{2}\right]}$$

(4) $$R=\frac{E\left[a^{T}XY^{T}b\right]}{\sqrt{E\left[a^{T}XY^{T}a\right]E\left[b^{T}YY^{T}b\right]}}=\frac{a^{T}C_{xy}b}{\sqrt{a^{T}C_{xx}ab^{T}a^{T}C_{yy}b}}$$

**Figure 2:**
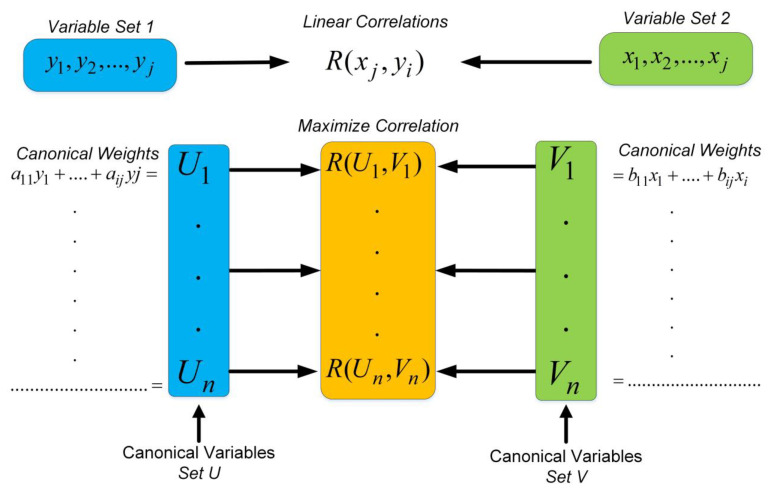
Schematic diagram of CCA with parameters.

A schematic representation of CCA with canonical variables, coefficients, and other parameters is shown in Figure 2 [31].

Pairs of canonical variables were obtained from a high to a low correlation coefficient value because of the CCA process. Various parameters were used to test the significance of canonical variable pairs obtained from the analysis. The first is the correlation coefficient, followed by Wilks’ lambda test. Wilks’ lambda test, which is computed over the correlation coefficient as given in Equation (5), provides preliminary information about the obtained canonical correlation coefficients and canonical variables, as well as preliminary information about their importance. Moreover, to examine the significance of the derived canonical variables, various test methods like F-static, degrees of freedom, and chi-square (*X^2^*) tests are available [32].

(5) $$⋀=\prod (1-{R_{i}}^{2})$$

While determining the variables affecting cognitive load in the programming process, the CCA approach is preferred for various reasons. CCA, which includes more than one dependent variable, goes beyond the multivariate regression analysis used when there is only one dependent variable. The advantages of CCA over traditional correlation analysis originate from its being based on a specific coordinate system. The CCA has the analytical solution in closed form. Moreover, CCA is invariant to scaling. Nonlinear methods require predefined kernel functions, and the correct choice of this kernel function and its related parameters directly affect the analysis’s success. Also, in nonlinear approaches, it is challenging to project back from the kernel space to the original feature space, which leads to difficulties in interpreting and visualizing the outputs of nonlinear approaches. In this context, the CCA was preferred because statistically significant and sufficient results were obtained.

### 2.2. Cognitive Load and EMIP Dataset

While analyzing the cognitive loads of programmers during programming processes, the EMIP dataset was used [33]. The EMIP dataset contains 216 individuals with different experience levels, genders, languages, and ages. It includes the eye movements of programmers during the process of two code comprehension tasks, and this comprehensive dataset is publicly available. The SMI RED250 eye-tracking device was used to create the EMIP dataset. The related eye tracking device has a sampling rate of 250 Hz and an accuracy value of <0.4°, and thanks to the high sample rate, it is also suitable for saccadic eye movements detection. When creating the EMIP dataset, two multiple-choice code comprehension tasks were presented to programmers [34]. These tasks are called the Rectangle and Vehicle tasks, respectively. The Rectangle task involves creating a class with four coordinate variables, a constructor, and methods for calculating the area, width, and height. The Vehicle task requires the development of a vehicle class with a set of variables, a constructor, and an acceleration function that can set the current speed variable. The primary method initiates and updates the development of a single object. Programmers must correctly determine the code functions in these operations using a set of multiple-choice options. Descriptive statistics of the characteristics of programmers for the EMIP dataset are presented in Table 1 [35].

The variables listed in Table 2 were used to create a meaningful relationship between the personal parameters of computer programmers and their cognitive load. According to related studies, there is a significant relationship between cognitive load and eye movements [36]. When creating the relevant dataset, parameters that model eye movement according to time were preferred as dependent variables [26,28]. The personal parameters of the computer programmers who created the EMIP dataset were used as the independent variables. In parallel with the literature, the dependent variables used in the related study consisted of meaningful attributes extracted from the eye movements of the programmers and the time to perform the given tasks. While creating a set of dependent and independent variables, we created a structure that provided a meaningful cause-and-effect relationship. Peak rate of eye movement, a dependent variable, represents rapid eye movements and is extracted from eye movements using adaptive signal-processing techniques. The other dependent variable used in this study was change in the position and radius of the pupil over time. The last dependent variable used in the study was the time to complete the related specific tasks of the programmers.

### 2.3. Linguistic Distance

In the EMIP dataset, some data are categorically defined, and transformation is required for multivariate analysis operations, such as the native language of programmers. Linguistic distance is the measure to which one language or dialect differs from another and is used for this transformation in this study [37]. To achieve this transformation, the results of a study involving many participants in the field of linguistics were used. The related research focuses on the challenges Americans face when learning foreign languages and aims to develop a quantitative measure of the distance between English and other languages. The transformation of linguistic distance from categorical to quantitative data is presented in Table 3. It shows the linguistic distance scores of sample languages from the EMIP dataset. According to the table, Chinese is the most distant, and Italian is the closest to English. The linguistic distance was expected to be important for this study because code comprehension tests and programming language command sets were created in English when developing the EMIP dataset.

### 2.4. Code Comprehension Tasks

**Figure 3:**
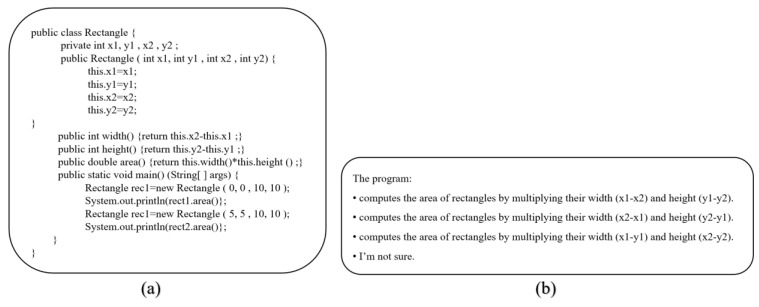
Rectangle code comprehension task. (**a**) Java code. (**b**) Multiple choice for Rectangle.

**Figure 4:**
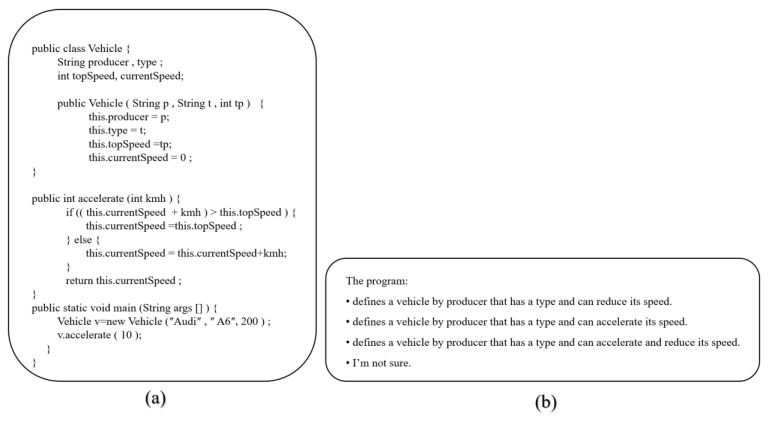
Vehicle class code comprehension task. (**a**) Java code for vehicle class. (**b**) Multiple choice for vehicle class.

Two code comprehension tasks were used to create the EMIP dataset. In the first task, the code related to the rectangular area was given, and the other was a task related to vehicle speed. Programmers are expected to look at the code and correctly determine its function among multiple options. The EMIP dataset was created by recording the eye movements of the programmers at these stages. Java code and multiple options for these two tasks are shown in Figure 3 and Figure 4, respectively [33].

### 2.5. Eye Movement Analysis

Eye movements are recorded with a sampling rate of 250 Hz, which is sufficient for detecting rapid eye movements. Saccadic eye movements are the leading eye movements associated with cognitive load. Saccadic eye movement is a rapid, conjugate eye movement in which the focus of the gaze moves from one area of the visual field to another. Rapid eye movements must be detected to determine saccadic eye movement. Therefore, changes in pupil coordinates were used in this study.

**Figure 5:**
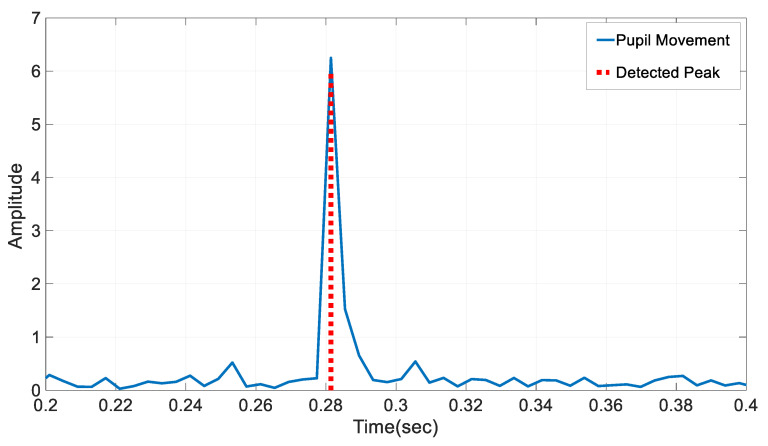
Movement of pupil and detected saccadic movement via *z*-score-based peak detection.

To determine saccadic eye movements from coordinate changes, a *z*-score-based peak detection method is preferred. The *z*-score concept is generally preferred in the normalization process of data, and its formula is given in Equation (6) [38].

(6) $$z=\frac{x-\bar{x}}{\sigma_{x}}$$

where $\bar{x}$ is the mean of the vector and $\sigma_{x}$ is the standard deviation of the vector. In the *z*-score-based peak detection algorithm, the influence of previous data on the vector is meaningful and valuable. Therefore, *z*-score-based peak detection can be used in various applications, such as physiological signal processing, sound processing, genetics, and fluid dynamics. Equations (7)–(9) are related to the *z*-score-based peak detection process. The mean and standard deviation (SD) of the specific part of the signal were calculated using Equation (7). The related approach is a peak detection approach that requires effective and meaningful thresholding [39].

(7) $${\bar{s}}_{i}=\frac{1}{l}\sum_{i}^{i+l}s_{i}\;\;\;\;\;{\;\;\;\;\;\;\;\;\;\;\;\;\;\;\;\;\;\;\;\;\;\sigma }_{s_{i}}=\sqrt{\frac{\sum_{i}^{i+l}{{(s}_{i}-{\bar{s}}_{i})}^{2}}{l-1}}$$

(8) $$s_{i}=i_{n}x_{i}+\left(1-i_{n}\right)\;\;\;\;\;\;\;\;\;\;\;\;\;\;\;\;s_{i-1}{\;z}_{i}=\frac{s_{i}-{\bar{s}}_{i-1}}{\sigma_{s_{i-1}}}$$

(9) $${th}_{s}=constant\;\;\;\;\;\;\;\;\;\;\;\;\;\;{\;y}_{i}=\left\{\begin{array}{c} 1\;if\;\left|z_{i}\right|\geq {th}_{s}\\ 0\;if\;\left|z_{i}\right|<{th}_{s} \end{array}\right.\;$$

where ${th}_{s}$ is the threshold value, l is the lag value. and $i_{n}$ is the influence factor, which can vary between 0 and 1. It defines the effect between the previous values of the signal and the new values. A low threshold allows noise in the signal to be identified as peaks, whereas a high threshold makes it difficult to identify true peaks. The lag value defines the size of the observation screen used for the peak detection analysis. A high lag reduces the sensitivity of the algorithm, whereas a low lag causes noise in the signal to be determined as the peak value. In this study, the parameters of the *z*-score-based peak detection algorithm for the detection of saccadic eye movements from pupil coordinates have been selected as follows: ${th}_{s}=5$, l=5, and $i_{n}=0.5$. These parameters were determined experimentally to optimize the signal-to-noise ratio and to prevent noise signals from being detected as peaks. An example output plot of the peaks obtained by the z-based peak detection method from eye movements in the EMIP dataset is shown in Figure 5.

**Figure 6:**
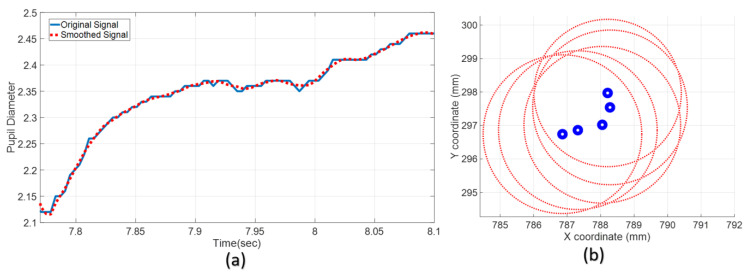
Pupil diameter and center coordinate. (**a**) Diameter change. (**b**) Coordinate change.

Figure 6 shows the changes in the pupil diameter and position over time. While the outer section of the generated circles represents the pupil diameter, the circle in the middle represents the pupil center coordinate. To reduce the noise in the raw measurement signals of eye movements and pupil diameters, Savitzky–Golay filtering, a polynomial fitting-based finite impulse response (FIR) smoothing filter, was used for pre-processing. To apply the Savitzky–Golay filter, the frame size and degree of the polynomial to be fitted must be defined, and a low-order polynomial was fitted through the data points using the least squares method [40]. In general, Savitzky–Golay filters improve the SNR ratios more than standard averaging FIR filters. Therefore, it is often preferred to reduce the noise ratio in physiological signals such as EEG, EKG, and EMG in the literature [41]. Eye movement data were made available after these signal-processing operations to build a meaningful correlation. 

## 3. Results

The canonical correlation analysis method was used to establish a meaningful and interpretable relationship between eye parameters affecting cognitive load and personal parameters of programmers. In the first stage, the data were normalized using the *z*-score normalization method as a pre-processing step. Partial correlation analysis was performed with the dependent and independent variables created within the scope of this study, according to Table 2, and the results are given in Table 4 and Table 5, respectively. The aim of the particle correlation analysis, which is performed as a preliminary analysis stage, is to reveal a meaningful correlation between and within variables [42].

As seen in the partial correlation results for the dependent variables, there is no strong relationship between these variables. Among the dependent variables, there is a weak relationship between eye movement SD and pupil radius change SD $(r_{42}=$ 0.244).

The highest correlations between independent variables, which are personal parameters of programmers, are calculated follows: between the age of programmer and time of experience in programming ${(r}_{71}=$ 0.66) and between expertise level in experiment language and expertise level in programming $(r_{85}=$ 0.613).

The correlation between independent and dependent variables was analyzed to establish a significant correlation between cognitive load and the personal parameters of programmers, and the results are shown in Table 6.

According to the results of the analysis, the dependent and independent variable groups with the highest correlation values are shown in Table 7 with *p*-values. As seen in the table, the relationship between these dependent and independent variables is statistically significant.

As shown by the results of the partial correlation analysis between the dependent and independent variables, there was a moderate correlation between the time it took programmers to complete the given tasks and their experience in the experimental language. In this study, moderate or weak correlation was determined according to the rule of thumb used to interpret the magnitude of the correlation coefficient [43]. A multivariate data analysis approach is required to improve the correlation coefficient and establish a more effective and meaningful relationship between the two sets of variables. For this reason, canonical correlation analysis was preferred, because it analyzes the effects on the variables in a meaningful manner and provides the parameters that will provide the maximum correlation between the two datasets. The correlation ratio between the datasets and the level of significance of the variables was examined using canonical correlation analysis. Canonical correlation analysis provided the canonical correlation coefficient between cognitive load parameters and programmers’ personal parameters, as well as canonical variable vectors and weights. The relationship between the two datasets was analyzed to determine the significance of the canonical correlation coefficients. The canonical correlation coefficients for the canonical variable vectors among the relevant datasets are listed in Table 8.

**Figure 7:**
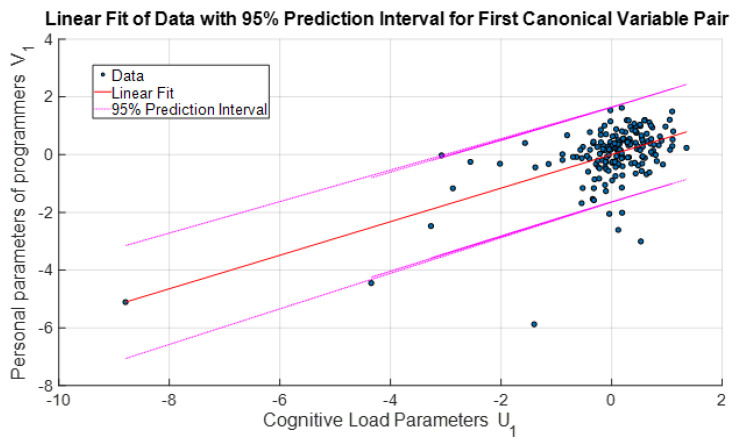
First pair (*U*_1_ and *V*_1_).

**Figure 8:**
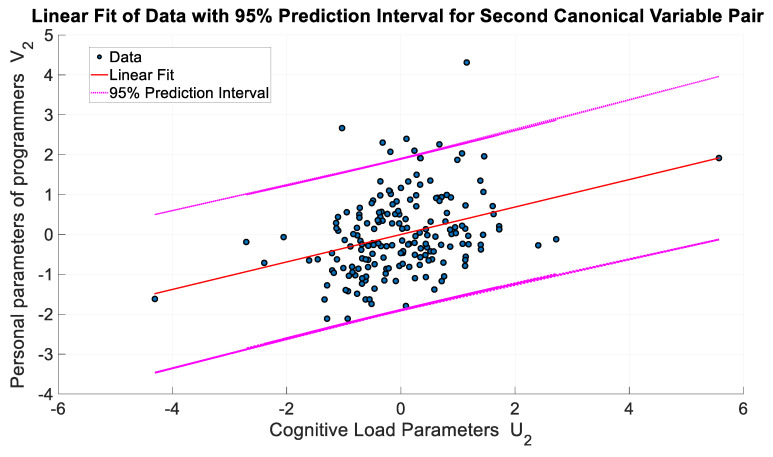
Second pair (*U*_2_ and *V*_2_).

When the particle correlation analysis and canonical correlation results of the relationship between cognitive load and the databases of programmers’ parameters are analyzed, it is seen that canonical correlation analysis yields more statistically significant results, as expected. In this way, the relationship between the dependent and independent variables was found statistically more meaningfully and became suitable for integrated analysis. Figure 7 and Figure 8 show the variation in the 1st and 2nd canonical variable vectors representing these relationships, respectively. When the related figures were analyzed, it was determined that the relationship between the first canonical variable pair was more linear and statistically more significant. Therefore, the first canonical variable pair was preferred for creating a mathematical model to define the relationship between programmers’ cognitive load and personal parameters. 

**Figure 9:**
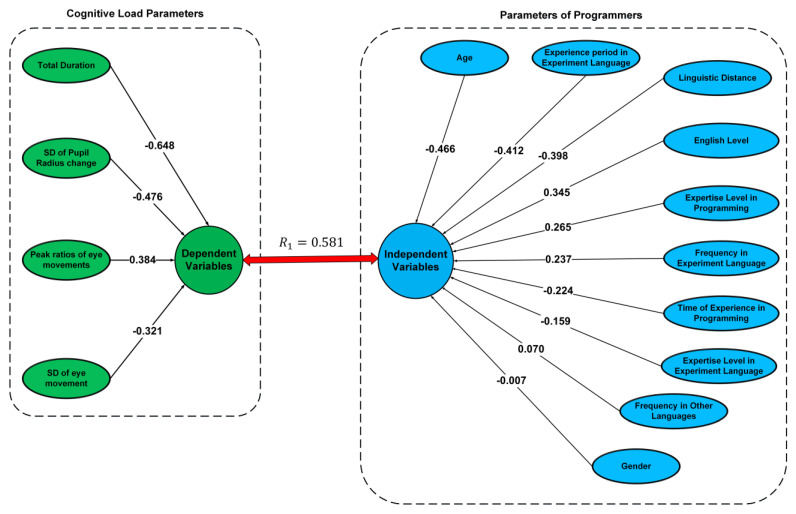
Path diagram of cognitive load and personal parameters.

When the relationship between the two canonical variable vector pairs is examined, it is clearly observed that the relationship of the first vector pair has the highest correlation and a more linear characteristic. In addition, this linear characteristic difference can be easily observed from the deviation in the distribution of data on the fitted optimal line. Using canonical weighting, which is statistically significant and has the highest correlation coefficient, a path diagram describing the relationship between cognitive load and the personal parameters of programmers was generated, as shown in Figure 9.

**Figure 10:**
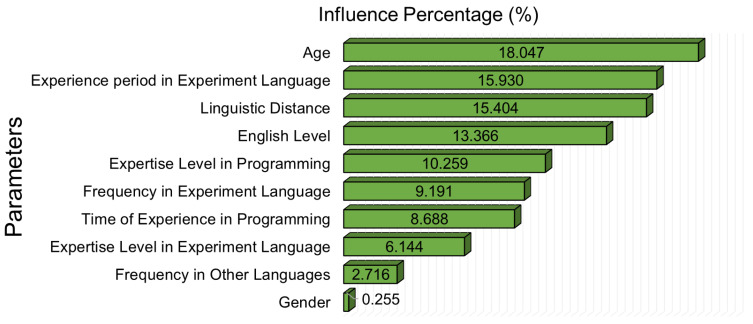
Factors influencing the cognitive load of programmers.

The amplitude of the canonical weights of canonical variables provides information regarding the influence of variables according to the datasets. Therefore, the relatively high amplitude of the weights provides information regarding the effect ratio between each dataset. To make the path diagram more meaningful using canonical weights, the percentage effects of the normalized canonical weights on the variables are shown in Figure 10. 

When the percentages of personal parameters affecting the cognitive load parameters of programmers were examined, it was determined that the highest effect was that of the age factor (18.047%), and the gender parameter had the lowest effect (0.255%). One of the important and novel results of this study is that the linguistic distance between a programmer’s native language and English has a significant effect on cognitive load (15.404%). When the canonical correlation analysis of the dataset is examined from another point of view, it is seen that there is a more balanced weight between the cognitive load parameters, which are as follows: peak ratio (20.9%), SD of eye movement (17.544%), SD of pupil radius (26.019%), and total duration (35.436%). 

## 4. Discussion

In this study, we have determined the parameters that affect the cognitive load of computer programmers during code comprehension tasks in percentage terms. We have used the EMIP database, a comprehensive database of eye movements and different personnel parameters of 216 participants [33,34].

We have transformed categorical variables, such as the native language of programmers in the relevant EMIP database, into meaningful and efficient quantitative variables using the linguistic distance approach [37]. We have extensively investigated the influence of linguistic distance on the cognitive load of computer programmers in this study for the first time in the literature with integrated sub-parameters.

To determine the rapid movements of the eye, the time-dependent changes in the pupil coordinates have been obtained, and the peak values for modelling the sudden movement of the eye have been determined with the *z*-score peak detection algorithm. We have used the Savitzky–Golay FIR smoothing filter to reduce signal measurement noise during data processing. We have assessed eye movements and problem-solving time as cognitive load parameters of programmers. The personal parameters of the programmers and the calculated cognitive load parameters have been determined as dependent and independent variables. First, we performed partial correlation analyses within and between these variables and tested the statistical significance of the results obtained. In the next step, we analyzed the effect of these variables on each other with the CCA approach to increase statistical significance and reveal more comprehensive results. As a result of the analysis, we have observed that not only the native language spoken but also the linguistic distance of the native language from English significantly affected the cognitive load. The results that we have obtained within the scope of this study support the results and suggestions of the studies examining the relationship between cognitive load and language [44]. 

We have determined the relationship between computer programming and spoken languages is essential according to cognitive load theory. Therefore, like the continuous exposure method used in foreign language education, teaching programming languages like spoken language can be meaningful and beneficial [45]. We have hypothesized that this approach could positively influence the process of learning a programming language in the future. 

We found that the age of the programmer had the highest effect on the cognitive load. Then, we determined that the duration of experience in the relevant programming language also had a significant effect on the programmer’s cognitive load. These outputs support the studies finding that age is an essential factor in cognitive load [46]. Studies conducted within the scope of analyzing learning processes have revealed that cognitive load increases with age [47].

One of the results of this study is that we have found that the frequency of using a different programming language had less effect on cognitive load. We have observed that specialization in or intensive use of a programming language with a different structure has a limited effect on the perception and interpretation of the problem encountered in another programming language. However, to support and generalize this inference, conducting comprehensive and specific studies that analyze the effect of different programming languages on cognitive load will be helpful. 

It is widely recognized that there are biological and social differences between the ways men and women perceive environmental stimuli [48]. However, current theories on cognitive load argue against the idea that these differences lead to precise and direct differences in working memory cognitive load. Cognitive abilities and performance are influenced by not only gender but also past experiences, education, and other factors that account for individual cognitive differences [49]. 

A comprehensive review study conducted in this context examined many neuroimaging studies to investigate gender differences in brain structures. Although researchers have found gender-related differences in some brain regions, these differences are insufficient to explain cognitive abilities [50].

Another comprehensive meta-analysis study investigating visual–spatial working memory in terms of gender differences has similar findings. The meta-analysis examined gender differences in visual–spatial working memory using a dataset of 180 effect sizes from 98 samples of healthy men and women aged 3 to 86 years. Statistical analyses of the study showed that these differences were too small to adequately explain gender differences in visual–spatial working memory abilities, particularly in mental rotation, where effect sizes may be relatively large [51].

As a result of this study, we have observed that gender difference has a limited effect on cognitive load in computer programming processes. One point not entirely comprehended in research on gender differences in cognitive load has been the deficiency of clear universal spatial or temporal patterns in males’ and females’ responses to external factors. In this study on computer programming and cognitive load, similar results to those in the literature have been obtained, and no significant correlation has been found between cognitive load and gender differences. The pattern of the relationship between gender differences and cognitive load will become more evident and meaningful with comprehensive studies with different stimuli over time.

This study has some limitations that should be emphasized. The programmers’ cognitive load or physiological fatigue levels were not measured physiologically or with various scales before performing the tasks. In the process of realizing the dataset, the programmers only looked at two code samples; both were object-oriented, so whether the obtained findings can be generalized to more algorithmic code or code in languages with different programming models may need to be clarified. As this was a multi-location study, minor differences in the experimental setup may have occurred despite using the same hardware in all locations, such as the same eye tracker and laptop. The use of eye tracking to measure cognitive load has important advantages, but in some cases, it can also have disadvantages. Studies conducted with eye-tracking systems can sometimes feel uncomfortable or artificial for participants. Therefore, they may cause changes in participant behavior that may affect the results of the studies.

## 5. Conclusions

The influence of personal parameters on the cognitive load in computer programming processes was examined in detail in this study. In this innovative study, the effect of code structures, which form the basis of computer programming, on cognitive load was investigated using the linguistic distance metric. The EMIP dataset, one of the most comprehensive datasets in this field, consisting of 216 programmers, was used in this study. Eye movements in two different code comprehension tasks and various personal parameters of the participants are available in the relevant datasets. In this study, the *z*-score-based peak estimation algorithm was used to determine rapid saccadic eye movement signals, an essential indicator of cognitive load. At the same time, the change in pupil diameter, another indicator of cognitive load change, was also analyzed. In the analysis, the statistical interpretation and validity of the results were tested in detail. The relationship between these comprehensive datasets was examined using canonical correlation analysis. The canonical weights obtained from the analysis were normalized, and the percentage effects of the parameters were calculated relative to each other. It was determined that the effect of linguistic distance analyzed within the scope of this study on cognitive load in the computer programming process is significantly important. It has been observed that the frequency of using a programming language other than that used in code comprehension tasks has a weak effect on cognitive load. Considering this result, the use of an alternate programming language has little effect on the cognitive load of programming in another language. It was determined that the effect of gender on cognitive load in the computer programming process was weak. In this regard, the cognitive load in computer programming processes can be independent of gender. It has been determined that the English level of programmers significantly impacts cognitive load in computer programming. 

## Figures and Tables

**Table 1:** Descriptive statistics for the EMIP dataset.

Parameters	Value
Number of Males	175
Number of Females	41
Mean (Age)	26.560
Standard Deviation (Age)	9.276
Mean (Time of Experience in Programming–Year)	2.259
SD (Time of Experience in Programming–Year)	3.342

**Table 2:** Independent and dependent variables.

Independent Variables	Dependent Variables
**1.** Age **2.** Experience Period in Experiment Language **3.** Linguistic Distance **4.** English Level **5.** Expertise Level in Programming **6.** Frequency in Experiment Language **7.** Time of Experience in Programming **8.** Expertise Level in Experiment Language **9.** Frequency in Other Languages **10.** Gender	**1.** Total Duration **2.** Standard Deviation of Pupil Radius change **3.** Peak Ratios of Eye Movements **4.** Standard Deviation of Eye Movement

**Table 3:** Linguistic distance of different languages from English [37].

Categorical Data (Language)	Quantitative Data (Language Score)
German	2.25
Italian	2.5
Portuguese	2.5
Spanish	2.25
Finnish	2.00
Turkish	2.00
Greek	1.75
Thai	2.00
Chinese	1.50

**Table 4:** Particle correlation analysis results for dependent variables.

Variables	1	2	3	4
**1**	1.000	0.003	−0.026	−0.013
**2**	0.003	1.000	−0.048	0.244
**3**	−0.026	−0.048	1.000	0.009
**4**	−0.013	0.244	0.009	1.000

**Table 5:** Particle correlation analysis results for independent variables.

Variables	1	2	3	4	5	6	7	8	9	10
**1**	1.000	0.051	0.148	−0.065	−0.230	−0.129	0.660	0.033	0.021	−0.144
**2**	0.051	1.000	−0.089	0.051	−0.203	−0.114	0.379	0.417	0.076	−0.032
**3**	0.148	−0.089	1.000	0.399	0.001	0.045	−0.105	0.054	−0.096	−0.099
**4**	−0.065	0.051	0.399	1.000	−0.027	−0.018	0.072	0.022	0.057	−0.100
**5**	−0.230	−0.203	0.001	−0.027	1.000	−0.353	0.447	0.613	0.397	0.055
**6**	−0.129	−0.114	0.045	−0.018	−0.353	1.000	0.027	0.600	0.062	0.155
**7**	0.660	0.379	−0.105	0.072	0.447	0.027	1.000	−0.139	−0.120	0.106
**8**	0.033	0.417	0.054	0.022	0.613	0.600	−0.139	1.000	−0.045	0.019
**9**	0.021	0.076	−0.096	0.057	0.397	0.062	−0.120	−0.045	1.000	0.091
**10**	−0.144	−0.032	−0.099	−0.100	0.055	0.155	0.106	0.019	0.091	1.000

**Table 6:** Particle correlation analysis results between independent and dependent variables.

		Dependent Variables			
Independent Variables	**Variables**	**1**	**2**	**3**	**4**
	**1**	0.300	0.318	−0.141	0.182
	**2**	0.404	0.183	−0.003	0.011
	**3**	−0.059	0.097	−0.156	0.159
	**4**	−0.078	−0.006	0.083	0.056
	**5**	0.111	0.058	0.060	−0.062
	**6**	−0.083	0.097	0.219	−0.136
	**7**	0.330	0.235	−0.051	0.091
	**8**	0.137	0.074	0.054	−0.084
	**9**	0.025	−0.009	0.086	−0.085
	**10**	0.039	−0.104	0.033	−0.052

**Table 7:** Particle correlation analysis results for dependent variables.

Independent Variables	Dependent Variables	Correlation Coefficient
Experience Period in Experiment Language	Total Duration	0.404 *
Time of Experience in Programming	Total Duration	0.330 *
Age	SD of Pupil Radius Change	0.318 *

Note: * *p* < 0.001.

**Table 8:** Correlation coefficient between canonical variables vectors.

Number of Canonical Variables	Canonical Correlation Coefficient
1	0.581 *
2	0.344
3	0.313
4	0.103

Note: * *p* < 0.001.

## Data Availability

The data presented in this study are available on reasonable request from the corresponding author.
